# A short note on predicted motifs in the *Thaparocleidus wallagonius* genome

**DOI:** 10.6026/97320630017940

**Published:** 2021-11-30

**Authors:** Kalpana Singh, Bhumika Chauhan, Anshu Chaudhary, Monica Misra, Bindu Sharma, Hridaya S Singh

**Affiliations:** 1 Laboratory of Molecular Parasitology, Department of Zoology, Chaudhary Charan Singh University, Meerut - 250004 (U.P.), India; 2 Molecular Taxonomy Laboratory, Department of Zoology, Chaudhary Charan Singh University, Meerut-250004 (U.P.), India; 3 Department of Zoology, Achrya Narendra Dev College, University of Delhi, India

**Keywords:** Thaparocleidus, wallagonius, sequence motif, genome, 18S rDNA, ribosome, drug discovery

## Abstract

*Thaparocleidus wallagonius* is a monogenean parasite and a fish-borne pathogen with a worldwide distribution. The genome for *Thaparocleidus wallagonius* is known. Therefore, it is of interest to report the DNA motif analysis data in the 18S rDNA of *Thaparocleidus wallagonius* collected from the fish Wallago attu in India. This data forms a framework for an in-depth analysis of the parasite biology and development, immune evasion strategies, virulence and long-term survival within the definitive host.

## Background:

Parasitic infections in fishes have been reported from different geographical regions worldwide. They aggravate a great threat to fish culture as fishes are important source of food in the developing countries [1]. Consumption of raw or uncooked fish is the main reason for the infection. This gives an opportunity for the parasite to remain into the host causing infections [2]. The genus Thaparocleidus was proposed by Jain (1952) for the species *Thaparocleidus wallagonius* (a monogenean parasite) isolated from the gills of Wallago attu at Lucknow, India (Bloch & Schneider) [3]. Genus Thaparocleidus is identified by the presence of both pairs of anchors (ventral pair are usually shorter than the dorsal pair) with roots of varying length, the appearance of the spot on the dorsal anchor, larval type of marginal hooks, and complete ventral bar [4]. It is known that changes to the ribosome core can affect the mechanism of translation initiation that is favored in the cell, potentially leading to specific changes in the relative efficiencies with which different proteins are made [5]. Genome data help understand biology to decipher the functioning of cells in organisms [6] using known Bioinformatics tools [7]. Therefore, it is of interest to report the sequence DNA motif analysis of 18S rDNA of *Thaparocleidus wallagonius* collected from the fish Wallago attu in India.

## Materials & Methods:

### Sequence:

The DNA sequence for the 18S rDNA of *Thaparocleidus wallagonius* was downloaded from the NCBI (National Centre for Biotechnology Information) database (https:/www.ncbi.nlm.nih.gov) with accession number KX364086.1.

### Motif analysis:

The motifs were analyzed using the GLAM2 Web tool and TRANSFAC Motif tool as shown in Figure 0(see PDF).

## Results and Discussion:

We found 6 motifs at 17 positions within the ribosomal genome of the parasite. rDNA is conserved and serves as a molecular machinery for protein synthesis. Data on motifs in the genome will help in blocking the activity of the parasite. Motif from positions 13-16 and 124-127 had a role associated with Glycosylation serving as ASN_GLYCOSYLATION (N-glycosylation site) (Figure 1A - see PDF). Motif cAMP_PHOSPHO_SITE was found at positions 120-123 (Figure 1B - see PDF). Motif CK2_PHOSPHO-SITE (Casein Kinase II phosphorylation site) was found on various positions i.e. from 26-29, 72-75, 149-152, 193-196, and 339-342 as shown in Figure 2. Motif MYRISTYL (N-myristoylation site) was found from positions 40-45, 268-273, and 276-281 (Figure 1C - see PDF). Motif PKC_PHOSPHO_SITE (Protein Kinase C phosphorylation site) was found at positions 65-67, 72-74, 145-147, and 293-295 (Figure 1D - see PDF). Motif TYR_PHOSPHO_SITE (Tyrosine kinase phosphorylation site) was also found at positions 84-92 and 225-231 (Figure 1D - see PDF). The role of these motifs has been widely studied in coding DNA till date. The coded functions includes protein folding and conformational stability, intracellular transport, antibiotic resistance and homeostasis processes, resistance to digestion by proteases, interaction with cell-surfaces, extracellular matrix proteins and other ligands, sexual commitment, inducing chemotaxis, DNA repair, cell cycle control, regulating the circadian rhythm and other cellular-processes, providing innate immune response, phosphorylation sites on human lamin and in nuclear lamina structural dynamics. This data is relevant in the understanding protein sequence, structure and function in drug discovery for the pathogen.

## Conclusion:

We report the presence of sequence motifs such as AMIDATION, ASN-GLYCOSYLATION, CAMP_PHOSPHORYLATION, CK2_PHOSPHORYLATION, N-MYRISTOYLATION and PKC_PHOSPHORYLATION sites in the T. wallagonius genome for further consideration in the understanding protein sequence, structure and function in drug discovery for the pathogen.
